# Speechlessness in Gilles de la Tourette Syndrome: Cannabis-Based Medicines Improve Severe Vocal Blocking Tics in Two Patients

**DOI:** 10.3390/ijms18081739

**Published:** 2017-08-10

**Authors:** Ewgeni Jakubovski, Kirsten Müller-Vahl

**Affiliations:** Department of Psychiatry, Socialpsychiatry and Psychotherapy, Hannover Medical School, Carl-Neuberg-Str. 1, 30625 Hannover, Germany; mueller-vahl.kirsten@mh-hannover.de

**Keywords:** Tourette syndrome, tics, nabiximols, blocking tics, dysfluency, cannabis

## Abstract

We report the cases of two young German male patients with treatment-resistant Tourette syndrome (TS), who suffer from incapacitating stuttering-like speech disfluencies caused by vocal blocking tics and palilalia. Case 1: a 19-year old patient received medical cannabis at a dose of 1 × 0.1 g cannabis daily. Case 2: a 16-year old patient initially received dronabinol at a maximum dose of 22.4–33.6 mg daily. Both treatments provided significant symptom improvement of vocal blocking tics as well as of comorbid conditions and were well tolerated. Thus, cannabis-based medicine appears to be effective in treatment-resistant TS patients with vocal blocking tics.

## 1. Introduction

Tourette syndrome (TS) is characterized by the presence of multiple motor tics and at least one vocal tic. TS is a relatively frequent condition with an estimated prevalence of about 1% [1,2]. Males are affected three-to-four times more often than females [2]. More severely affected patients usually exhibit more complex tics including coprophenomena, echophenomena such as imitating gestures (echopraxia) and words or phrases (echolalia), and paliphenomena such as phonic blocking and repetition of own words and syllables (palilalia) [3]. The prevalence of blocking motor and vocal tics is unknown, but blocking phenomena in TS seem to be quite rare and occur in more severely affected patients [4]. However, severe palilalia and vocal blocking might in some rare cases highly affect the fluency of speech and resemble the phenomenon of stuttering, resulting in significant social problems [4]. Due to this resemblance and a clinically observed overlap of tics and speech pathology symptoms, a number of TS patients might be misdiagnosed as suffering from a speech pathology [5]. Although phenotypically similar, tic-induced disfluency in speech was shown to be quite different from speech pathologies such as stuttering and cluttering [6,7]. It is therefore quite possible that TS patients who are misdiagnosed as stutterers will not profit from typical speech therapy and should rather receive a tic-specific treatment [8].

The state-of-the-art treatment options for tics include behavioral therapy and antipsychotic medication, which are known to cause significant side effects and to be insufficiently or not at all effective in a substantial number of patients with TS [9,10]. Thus, there is a clear demand for new and more effective treatment options with fewer side effects. After first anecdotal reports of successful self-medication with cannabis have been published [11,12], the central endocannabinoid system (ECS) was proposed as an alternative mechanism of drug action [13]. Accordingly, cannabis-based medicine (CBM) such as dronabinol (delta-9-tetrahydrocannabinol, THC) [14,15] and nabiximols [16,17]—which contains THC and cannabidiol (CBD) at a 1:1 ratio—have been suggested as new treatment strategies for patients with TS.

Here we report two cases of TS patients who suffered in particular from disabling vocal blocking tics and palilalia. After having failed several state-of-the-art treatments, treatment with different CBMs were started to improve speech fluency, social impairment, and patients’ quality of life. Improvement was assessed through clinical evaluation of the symptom presentation at the follow-up appointments. Percent improvement was a subjective estimate reported by the patients.

## 2. Cases

### 2.1. Case 1—A Treatment with Medical Cannabis

Pregnancy in this 19-year-old German male with no family history of tic disorders was complicated by false labor starting at month 5. He was born on week 36 (weight: 2800 g, height: 50 cm) and was kept at the newborn ward for a 10-day observation period. His further development followed normal patterns and he learned to speak at 3 years. However, from this age on, he suffered from speech pathology issues including universal dyslalia and dysgrammatism. He received appropriate speech therapy for both conditions, resulting in symptom improvement. However, over the course of the treatment the patient started exhibiting a new symptom resembling a stuttering. Despite being directly targeted by speech therapy, this symptom showed no signs of improvement. At age seven, a first motor tic—mouth opening—was observed, followed by first simple vocal tics in the form of coughing and producing a “hm” sound. The patient’s speech therapist suspected a connection between the stuttering-like symptom and the patient’s tics and referred him to our specialty TS clinic for further evaluation, where he was initially seen in 2005 at the age of eight. At his initial presentation, the patient exhibited typical motor tics including tensing of hands, stomping, blinking, nodding, tensing of the upper body, and kicking. In addition, typical simple vocal tics such as animal noises were observed. Clinically relevant comorbidities were seen neither at the first nor at follow-up visits. The speech impairment was identified as a complex vocal tic in the form of a combination of early onset of palilalia and a vocal blocking tic, which phenotypically resembled a stuttering. Over the course of his childhood and adolescence, the patient remained in our outpatient clinic and was seen on a regular basis for medical management and further clinical consultations. The course of the vocal blocking tic followed the typical waxing and waning time course also observed in his other tics. Three speech therapies over three years remained without any improvement, which further validated the diagnosis of vocal blocking tics instead of primary stuttering. However, over the course of puberty, the patient’s tics and in particularly vocal blocking phenomena deteriorated further. As a result, his school performance decreased and he was at risk of having to repeat the eighth grade. Starting at age 9, the patient received a number of different medical treatments consisting of atypical antipsychotic medications including tiaprid (up to 900 mg/day), sulprid (up to 800 mg/day), risperidone (up to 1 mg/day), and aripiprazole (up to 30 mg/day). Over the years, the patient best profited from tiaprid treatment, although the effect was only mild. Combination with other antipsychotics did not lead to significant symptom improvement. Subsequently, tiaprid was increased to 900 mg. However, at age 14 dose needed to be lowered due to significant weight gain and 3 years later again (to 300 mg), when he developed acute dyskinesia. Finally, at the age of 19 years, the vocal blocking tic was by far the most disturbing tic and the patient was unable to hold a normal conversation for over a year. It could take him more than one minute to say one single word. The speech impairment depended highly on the situation: speaking to family members and friends was easier and more fluent, while speaking to strangers was barely possible. Understandably, these symptoms had a devastating influence on the patient’s everyday life, in particular social situations and performance at school. As a result of these difficulties, the patient withdrew from any social situations and reduced any outside activities, becoming more and more homebound. Academic performance was so poor that the patient was in danger of dropping out of high school without a degree.

At that time, the patient made the decision for treatment with CBM. As his insurance company had refused coverage of therapy costs with nabiximols, less expensive treatment with medical cannabis (based on a permission by the German federal opium agency) was initiated at a dose of 0.1 g vaporized medical cannabis (Bedrocan^®^, Veendam, The Netherlands), containing 22% THC and 1% CBD) once daily and was increased to 0.6 g/day. At the 8 months follow-up and subsequent visits, the patient presented very much improved, he was able to converse with the doctors fluently, and he reported to be able to speak nearly fluently in most situations. Improvement also occurred in other tics, such as head nodding. The patient reported an effect onset at 5–10 min after administration that lasted typically up to one hour and a half. Although the drug effect dissipated after that, he usually experienced a significant tic reduction of about 70% including the blocking speech tics and a feeling of “being calmer” throughout the whole day. Over the course of the first few weeks of treatment, the patient used to experience a “high” after administration that completely disappeared later in treatment. No further side effects were reported.

### 2.2. Case 2—A Treatment with Dronabinol

This 16-year-old German male had no family history of tic disorders. He was delivered via caesarean-section and reached his developmental milestones in a timely manner. First problems with speech fluency started as early as age 3 and mostly resembled a stuttering-like phenomenon. Typical motor tics started at age 6, including multiple facial tics and arm movements as well as simple vocal tics such as animal noises (grunting). Over the course of his childhood and early adolescence, the patient received several speech therapies (altogether over 10 years) under the diagnosis of primary stuttering including inpatient treatment in a specialty speech clinic, which did not result in significant symptom improvement. At age 14, treatment with tiaprid (up to 200 mg/day) was initiated by his neurologist, which initially improved his symptoms, but came at the cost of serious weight gain of about 20 kg and gynecomastia. Later that same year, the patient presented to the department of pediatric audiology at our university clinic and was referred to our specialty TS clinic due to his motor and vocal tics. At first consultation, he suffered from several motor tics including head shaking, eye movements, abdominal tensing, arm movements, and finger sniffing, simple vocal tics including the production of a “hm” sound and exhaling and complex vocal tics including palilalia, vocal blocking, and a change of melody and rhythm of speech (which were most disturbing). For the first time, the diagnosis of TS was made. Apart from tics, he suffered from a range of comorbid symptoms including rage attacks, sleeping problems, tic-related anxiety and shame about speaking in public, depressed mood, and obsessive-compulsive symptoms (OCS) (ordering of pencils, not just right feeling, and rumination) resulting in difficulties concentrating. At that time, he could no longer fully-participate at school due to his increasingly impairing complex vocal tics. We therefore started treatment with aripiprazole at a dose of 2.5 mg, which was reported to make tics and OCS worse and to cause significant sedation as soon as the dose was raised up to 5 mg. It was subsequently discontinued by the patient, who refused any further medication. Therefore, our next treatment attempt included behavioral treatment with habit reversal training, however, this did not result in sufficient symptom improvement either. Meanwhile, the patient suffered from worsening mood swings, rage attacks, and attention problems, altogether resulting in further deterioration of academic performance and social problems followed by social withdrawal and was at danger of having to repeat the grade.

Therefore, we decided in favor of treatment with vaporized dronabinol (up to a maximum dose of 3 × 8 drops daily (=16.8 mg dronabinol), 1 drop = 0.7 mg dronabinol) despite the patient’s young age of 16 years. At the 8 months follow-up appointment, the patient reported significant improvement with only minimal vocal blocking tics. Although several tics could still be observed including head jerks, arm movements, blinking, and blocking of movements, they were significantly improved. The patient’s speech was mostly fluent and fully understandable, and only somewhat chopped. Further significant clinical improvement had occurred in impulsive behaviors, rage attacks, anxiety, OCS including not just right feeling, as well as in academic performance and social withdrawal.

At the one year follow-up appointment, the patient presented with a markedly worsened overall symptomology including severe school anxiety, frequent rage attacks, impulsivity, inattention, self-injurious behavior by cutting in the abdominal area, and increased OCS. School attendance needed to be paused for about three months due to rage attacks and conflict in school environment. Upon closer examination, the patient admitted having stopped taking dronabinol and instead having self-medicated with alcohol. Only after the patient had been directed into individual psychotherapy to address his drinking problems and after cessation of all drinking behaviors, treatment with dronabinol was restarted at a maximum dose of 8–12 drops four times daily (22.4–33.6 mg dronabinol) without side effects. Eight months later, the patient reported an overall tic improvement of about 70%, an improvement of his speech fluency by about 25%, feeling more relaxed and better focused. This was very relevant for the patient’s everyday life because it made communication with others possible and even enabled him to hold presentations in front of the class, which resulted in a significant improvement of school grades.

## 3. Discussion

We report about two young men with TS characterized by significant speech disfluencies caused by complex vocal tics such as blocking and palilalia. In both patients, speech problems started in early childhood and were initially diagnosed as primary stuttering. Reassessment in our specialized TS clinic identified the symptoms to represent complex vocal tics in form of vocal blocking for the following reasons: (i) speech problems followed typical waxing and waning as seen also in patients’ other motor and vocal tics; (ii) state-of-the-art speech therapy failed to improve speech problems; (iii) speech problems partially improved using anti-tic medication with tiaprid; and (iv) treatment with CBM resulted in comparable improvements of both speech problems and other motor and vocal tics. However, in these two patients treatment with CBM—either medical cannabis (case 1) or dronabinol (case 2)—resulted in significant improvement not only of simple and complex motor and vocal tics, but also in the overall symptomology including comorbid conditions and most importantly significantly improved patients’ quality of life including their social contacts and performance at school without side effects. Our results are in line with data from recent open uncontrolled case studies [12,13,16,18,19] and two small controlled clinical trials [14,15] reporting beneficial effects of different CBM including cannabis, dronabinol, and nabiximols in patients with TS. Furthermore, our findings further corroborate available reports suggesting that CBM may improve not only tics, but also psychiatric comorbidities including OCS [13,20], self-injurious behavior [11], and impulse control [20] in this group of patients.

Due to significant waxing and waning usually observed in tic disorders, in single cases it is always difficult to link symptom improvement reliably to a medication effect. Nevertheless, a case can be made for individuals who are severely affected by their tics for an extended period of time with a very low chance for spontaneous improvement, who then experience a substantial improvement following the administration of a new treatment. We believe this is the case for the two patients presented in this report. The medication effect could particularly be observed in the case of the second patient, who experienced a significant relapse during the time of his noncompliance. As in other case studies, a placebo effect cannot be distinguished from the medication effect. However, it would be quite unlikely to see a pronounced placebo effect in otherwise treatment-resistant patients. Another limitation is that little is known about the subgroup of patients with vocal blocking tics, who are phenotypically quite different from patients with a more common tic presentation. To date, too little can be said about the existence of this subgroup and therefore about the generalizability of our findings.

With these case reports we would like to highlight (1) the phenomenon of vocal blocking tics in patients with TS, which might be misdiagnosed as primary stuttering even in those patients who have been diagnosed with TS; (2) that in our experience speech therapy is ineffective in the treatment of severe vocal blocking tics; (3) that medical treatment with antipsychotics often fails to significantly improve vocal blocking tics; and (4) that CBM such as medical cannabis and nabiximols might be a valuable treatment option for these patients.

In summary, we suggest that greater awareness should be raised for the existence of tic-related stuttering-like symptoms and efforts should be made to avoid misdiagnoses, because severe vocal blocking tics regularly cause significant social impairment. If other therapies fail to improve this form of complex vocal tics, treatment with CBM should be taken into consideration.
